# Data driven precision medicine: who is the driver?

**DOI:** 10.18632/oncotarget.27860

**Published:** 2021-02-16

**Authors:** Sebastian Klein, Reinhard Büttner

**Keywords:** pleomorphic dermal sarcoma, sarcoma, computational biology, computational pathology, precision medicine

## Precision medicine: discovering genetic alterations and tumor-microenvironmental cues to propose treatment options

Treatment options for patients with lung- and skin cancers, and various other entities, have improved dramatically within the last decade [1–4]. Here, the discovery of targetable alterations, as well as a molecular- and cellular understanding shaped novel therapeutic approaches. However, these advances rely on large scale-, multi-centric- and multi-omics approaches [5, 6]. While the accumulation of data appears fundamentally important, the careful examination and even more evidently, integration and interpretation of these results is key.

**Figure 1:**
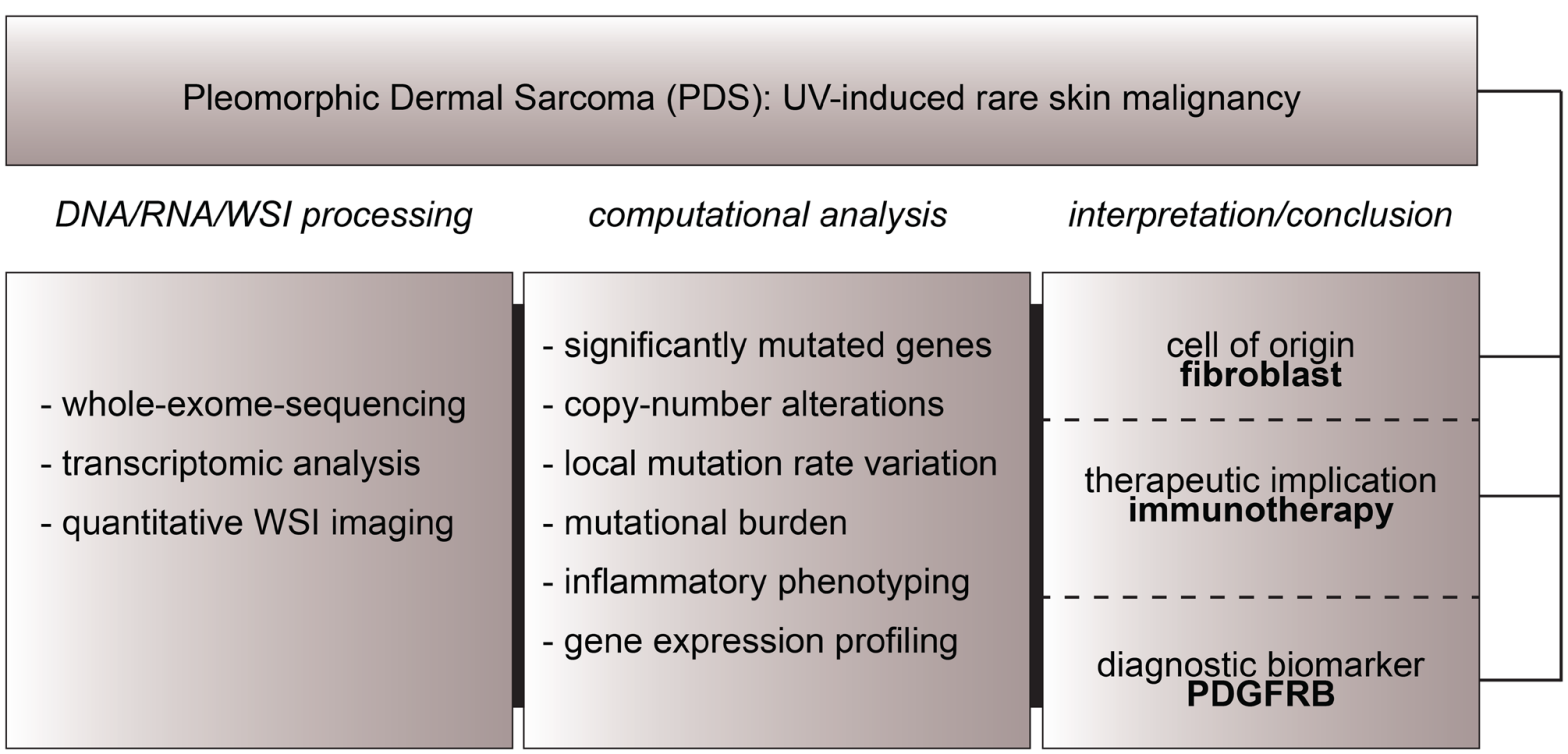
Study approach of our integrative analysis of pleomorphic dermal sarcoma. Whole-exome-sequencing, DNA/RNA analysis as well as whole-slide-image (WSI) analysis led to the discovery of a fibroblastic cell of origin in PDS, therapeutic implications- as well as a diagnostic biomarker for this entity.

Recently, we published our integrative analysis of a rare, UV-induced skin tumor called Pleomorphic Dermal Sarcoma (PDS) [7]. Within this study (Figure 1), we analyzed whole-exome-sequencing data, transcriptomic data, as well as imaging data to i.) propose a fibroblastic, mesenchymal cell of origin and to reveal ii.) treatment options, including immune-checkpoint inhibition, as well as iii.) PDGFRB as a potential diagnostic biomarker for PDS, complementing previous studies [8, 9].

While these results are especially promising for patients suffering from advanced PDS, they can also be used as a showcase for the necessity of a broad genetic analysis, also applied on rare tumor entities.

## Precision medicine: deep learning and what images can tell us

Despite DNA/RNA- sequencing approaches, the broad application of deep learning to uncover therapeutically targetable alterations in solid cancers from regular H&E images is emerging [10]. We also recently published an HPV-prediction score (HPV-ps) that can identify individuals with HPV-associated oropharyngeal squamous cell carcinomas (OPSCC) [11]. This study appears especially encouraging, as the combination of the proposed HPV-ps together with the p16-status outperformed the gold-standard of combined HPV-DNA and p16-status, which may inform future studies to reveal biomarkers for de-escalation trials within this disease. Importantly, the algorithm was highly predictive for outcome of patients and may help to tailor therapies of individual patients more precisely. In addition, we have published an approach of applying deep learning as a sensitive screening tool for the pro-tumorigenic bacterium H. *pylori*, the most important risk factor for gastric cancer, underlining a role of these technologies in preventing cancer development by improving diagnostic sensitivity [12].

## Precision medicine: a necessity for centralization

One may argue that the true challenge is to build diagnostic centers integrating information from genomics, transcriptomics, and deep learning algorithms on both DNA/RNA data, as well as whole-slide images (WSI) from tumors within their micromilieu. Evidently, this can only be guaranteed if forces are combined and data is shared among disciplines, and among treatment- and diagnostic centers. It appears unlikely, that this is possible without centralizing forces. Although on the contrary, economically- and potentially political reservations may lead to roadblocks within these developments. Thus, we must carefully answer the question: “*How do we improve the discovery of treatment options and diagnostic procedures for every single (cancer) patient, no matter where he or she is diagnosed?*”
